# Protein Lipidation Types: Current Strategies for Enrichment and Characterization

**DOI:** 10.3390/ijms23042365

**Published:** 2022-02-21

**Authors:** Rong Wang, Yong Q. Chen

**Affiliations:** 1 Wuxi School of Medicine, Jiangnan University, Wuxi 214122, China; 7170112036@stu.jiangnan.edu.cn; 2 School of Food Science and Technology, Jiangnan University, Wuxi 214122, China

**Keywords:** protein lipidation, polyunsaturated fatty acid, proteomics, mass spectrometry

## Abstract

Post-translational modifications regulate diverse activities of a colossal number of proteins. For example, various types of lipids can be covalently linked to proteins enzymatically or non-enzymatically. Protein lipidation is perhaps not as extensively studied as protein phosphorylation, ubiquitination, or glycosylation although it is no less significant than these modifications. Evidence suggests that proteins can be attached by at least seven types of lipids, including fatty acids, lipoic acids, isoprenoids, sterols, phospholipids, glycosylphosphatidylinositol anchors, and lipid-derived electrophiles. In this review, we summarize types of protein lipidation and methods used for their detection, with an emphasis on the conjugation of proteins with polyunsaturated fatty acids (PUFAs). We discuss possible reasons for the scarcity of reports on PUFA-modified proteins, limitations in current methodology, and potential approaches in detecting PUFA modifications.

## 1. Introduction

Proteins play indispensable roles in maintaining cell survival, and their functions are often regulated by post-translational modifications (PTMs), in which proteins are proteolytically cleaved or enzymatically conjugated with modifying groups. Various enzymes, including kinases, phosphatases, transferases, and ligases, catalyze approximately 500 discrete PTMs of a diverse set of proteins [1,2]. PTM of proteins occurs at all stages of human life, and abnormal PTM often leads to various diseases [3,4,5,6,7].

Well-studied PTMs include protein glycosylation, methylation, hydroxylation, amidation, phosphorylation, acetylation, and ubiquitination [2,8]. Protein lipidation is perhaps not as extensively studied as protein phosphorylation or acetylation even though it is no less significant than other modifications [9,10,11,12,13]. Various lipids or lipid metabolites can be covalently attached to proteins, and this PTM is accordingly called under different names, including protein lipidation [7], fatty acylation [14], and lipid modifications of proteins [15]. Nearly 20% of all proteins are known to be lipid modified [16], which is relatively rare, resulting in the detection difficulty and need of enrichment techniques for characterization.

There exist many technologies to characterize lipidated proteins by taking full advantage of the proteins’ characteristics, including spectroscopic methods, such as nuclear magnetic resonance (NMR) spectroscopy [17] and circular dichroism (CD) spectroscopy [18] (according to membrane protein structure and dynamics), crystallography [19,20] (according to lipidated protein dimensional structure), mass spectrometry (MS) [7] (according to lipidated protein fragment-ion characteristics), and so on. However, given the wide structural variability of lipid moieties of lipidated proteins, highly sensitive and specific methods for its detection are required [21]. Adequate enrichment followed by MS will be a more effective strategy.

There are many related reviews about protein lipidation, however, these reviews focused on the most common lipid-modifications, such as S-palmitoylation. For instance, Xu et al. reviewed S-palmitoylation and its significance in protein regulation, cell signaling, and diseases [7]. Here, we summarize the various types of protein lipidation, with an emphasis on polyunsaturated fatty acid (PUFA) modification, and methods used to detect them.

## 2. Types of Protein Lipidation

To date, studies have shown that proteins can be modified by at least seven types of lipids, including fatty acids, lipoic acids, isoprenoids, sterols, phospholipids, glycosylphosphatidylinositol (GPI) anchors, and lipid-derived electrophiles (LDEs).

### 2.1. Fatty Acylation

#### 2.1.1. S-palmitoylation

Protein S-palmitoylation refers to the covalent attachment of palmitic acid (C16:0) to the side chain of a cysteine residue on a protein through a thioester bond (Table 1). Palmitoylation is a reversible modification in organisms with specific enzymes to catalyze the addition or removal reaction [22,23], discovered in 1960s [24,25,26,27]. S-palmitoylation is dynamically regulated by palmitoyl acyltransferases (PATs, also known as ZDHHC-PATs) and acyl protein thioesterases (APTs), and the conserved zinc-finger Asp-His-His-Cys (ZDHHC) motif in the functional region of these enzymes is necessary for this process. PATs attach palmitoyl-CoA to proteins, and APTs perform depalmitoylation. For example, palmitic acid is added onto the SCRIB protein by the zinc finger DHHC-type palmitoyltransferase 7 (ZDHHC7) [28] and removed by lysophospholipase 2 (LYPLA2, also known as APT2) [29]. In some cases, however, proteins can be directly bound to palmitoyl-CoA and undergo PAT-independent auto-palmitoylation [30]. To date, a family of 24 mammalian PATs has been identified [7,31]. The “SwissPalm” database shows that > 10% of the human proteome is susceptible to S-palmitoylation [32,33], in which >600 substrates have already been fully or partially characterized [34,35,36]. Proteins that can be S-palmitoylated include NRas proto-oncogene, GTPase (NRAS) [37], β_2_ adrenergic receptors (β_2_ARs) [38], Fas cell surface death receptor (FAS) [39], BCL2-associated X, apoptosis regulator (BAX) [40], inositol 1,4,5-triphosphate receptor type I (ITPR1) [41], junctional adhesion molecule 3 (JAM3) [42], and SRC proto-oncogene, non-receptor tyrosine kinase (SRC) [43].

#### 2.1.2. N-palmitoylation

N-palmitoylation is classified into N-terminal palmitoylation and N^ε^-palmitoylation according to the position of the modification in the protein (Table 1). In N-terminal palmitoylation, palmitic acid is linked to the amino group of the cysteine residue at the N-terminus of substrate proteins, whereas in N^ε^-palmitoylation, palmitic acid is covalently attached to the ε-amino group of the lysine residue at the N-terminus via an amide bond. The biological significance of N-terminal palmitoylation has been reviewed before [31,44,74]. A unique dual palmitoylation in the N-terminal region of the human LIM domain kinase 1 (LIMK1) controls the targeting of this protein to the spine and contributes to the activation of the protein by membrane-localized p21-activated kinase (PAK) [74]. N-terminal palmitoylation has also been detected in Sonic Hedgehog (SHh) proteins [75] and shown to be catalyzed by Hedgehog acyltransferase (HHAT) [76]. Additionally, Sirtuin (SIRT) has been reported to be modified by N^ε^-palmitoylation [45,46,77,78].

#### 2.1.3. O-palmitoylation

Palmitic acid can be irreversibly linked to the side chain of serine residues in proteins via ester bonds in organisms without specific enzymes removing the attached lipid chain (Table 1). Currently, only a few proteins are known to be O-palmitoylated. One of them is histone H4, which can be O-palmitoylated at Ser-45 by an enzyme called lysophosphatidylcholine acyltransfer ase 1 (LPCAT1) [47]. Interestingly, O-palmitoylation at the threonine residue in the C-terminal of the spider venom neurotoxin PLTX-II has been reported, and it is thought to regulate the toxin activity in blocking presynaptic voltage-gated Ca^2+^ channels [48].

#### 2.1.4. N-myristoylation

Similar to N-palmitoylation, N-myristoylation is also categorized into two major classes—N-terminal myristoylation and N^ε^-myristoylation (Table 1). N-terminal myristoylation is the attachment of myristic acid (C14:0) to a protein N-terminus with a glycine residue through an amide linkage. This reaction is catalyzed by N-myristoyltransferases (NMTs), which recognize the GXXXS/T signature sequence (where X is any amino acid) in substrate proteins [49]. Human proteomic studies have suggested that > 100 proteins are N-myristoylated [79], such as A-kinase anchoring protein 12 (AKAP12) [77], SRC [78], protein kinase AMP-activated non-catalytic subunit beta 1 (PRKAB1) [80], FMR1 autosomal homolog 2 (FXR2) [81], and hexokinase 1 variant in mammalian spermatozoa (HK1S) [82], underscoring the vital roles of N-myristoylation [83]. N^ε^-myristoylation refers to the attachment of myristic acid to the ε-amino group of a lysine residue in substrate proteins. The enzyme(s) that catalyze this PTM is (are) not known; however, N^ε^-myristoylated proteins can efficiently be deacylated by SIRTs [50,51,52].

#### 2.1.5. Acylation of Other Saturated Fatty Acids

In addition to palmitoylation and myristoylation, proteins can be lipidated with other types of long-, medium-, or short-chain saturated fatty acids (Table 1). Studies have shown that influenza virus hemagglutinin can be S-acylated by stearate (C18:0) [53,54], Ghrelin can be O-octanoylated (C8:0) [55,56]. In 2009, the Zhao group released a bioinformatic tool named PTMap [84], which helps identify various acylations, including Lys propionylation, butyrylation, hydroxyl-fatty acid modification, lactylation, bicarboxylic acid modification, and benzoylation. Numerous studies have discovered >500 histone modification sites for above listed modifications, greatly expanding the understanding of PTM and histone modifications [85].

#### 2.1.6. Acylation of Unsaturated Fatty Acids

Physeterylation (C14:1n9) is detected in the retina, heart, and liver [86] and on SRC family kinases [87]. Myristoleoyted (C14:1n5) proteins have also been found [88,89]. WNT proteins are O-palmitoleoylated with palmitoleic acid (C16:1n7) on their conserved serine residue by the O-acyltransferase Porcupine [57,58,90], and the palmitoleic acid of an O-palmitoleoylated WNT protein is removed by Notum [59]. Oleic acid (C18:1n9) modification has been reported on the lysine residue of the lens integral membrane protein aquaporin-0 and plays an important role in targeting the substrate protein to membrane domains in the bovine and human lens [60] (Table 1).

### 2.2. N-lipoylation

Lipoylation, the attachment of lipoic acid to a lysine residue in proteins (Table 1), is a relatively rare PTM associated with human metabolic disorders, cancers, and mental diseases [91]. The deacylase of lipoylated proteins is SIRT [62,63]. In mammals four multimeric metabolic enzymes—pyruvate dehydrogenase (PDH), α-ketoglutarate (KDH), branched-chain keto acid dehydrogenase E1 subunit alpha (BCKDHA), and glycine cleavage system (GCV)—are lipoylated and participate in the TCA cycle [91,92]. This modification confers a “swinging arm” conformation to the protein structure for enzymatic reactions [91].

### 2.3. S-prenylation

S-prenylation is the attachment of isoprenoids to a cysteine residue in proteins [65]. Up to 2% of the total cellular proteins in mammalian cells are prenylated [93]. This modification occurs on one or more sidechains of a cysteine residue located at or near the C-terminus of the protein substrate. Most S-prenylated proteins contain a CAAX motif at their C-terminus, where the As are aliphatic amino acids and the X can be any amino acid [64]. Based on the properties of the X residues, S-prenylation is categorized into two major types. If the X is a leucine or any other small residue (alanine/serine/methionine), a 20-carbon geranylgeranyl group is attached to the C-terminus of the protein substrate (i.e., S-geranylgeranylation). Otherwise, a 15-carbon farnesyl isoprenoid lipid is attached (i.e., S-farnesylation) [65]. The enzyme that catalyzes protein S-farnesylation is called farnesyltransferase (FTase), whereas S-geranylgeranylation is catalyzed by geranylgeranyltransferase type I (GGTase-I) (Table 1) or GGTase-II (also known as RabGGTase due to its specificity for Rab proteins) [90]. Inhibitors of FTase and GGTase-I are used to target Ras prenylation, especially for KRas proto-oncogene, GTPase (KRAS), which is frequently mutated in many types of cancers [94,95].

### 2.4. C-terminal Phosphatidylethanolaminylation

C-terminal phosphatidylethanolaminylation is the attachment of phosphatidylethanolamine (PE) to the amino group of a C-terminal glycine (Table 1). Microtubule-associated protein 1 light chain 3 alpha (LC3), a well-known protein associated with autophagy, is phosphatidylethanolaminylated [66,67].

### 2.5. C-terminal Cholesterolyation

C-terminal cholesterolyation is observed in Hedgehog (HH) family proteins and refers to the conjugation of cholesterol to the C-terminus of these proteins via an esterified linkage with the hydroxyl moiety of the cholesterol through an autocatalytic reaction (Table 1). The HH family plays fundamental roles in long-range embryonic signal transduction pathways [96]. HH proteins can undergo two types of modification, namely C-terminal cholesterolyation and N-terminal palmitoylation, which are both critical for the activities of HH proteins [68,69,97].

### 2.6. C-terminal GPI Anchoring

Glycosylphosphatidylinositols (GPIs) are synthesized within the ER membrane through successive addition of a monosaccharide, acyl group, and phosphoethanolamine residue to phosphatidylinositol, consequently forming complex glycolipids. These glycolipids can be covalently attached to the C terminus of proteins by a GPI transamidase complex [70] and removed by phosphatidylinositol-specific phospholipase C (PI-PLC) [71] (Table 1). Approximately 1% of eukaryotic proteins are GPI-anchored [98] and participate in many biological processes, including cellular communication, signal transduction, antigen presentation, oncogenesis, malaria, and neurodegenerative prion diseases [99,100,101,102].

### 2.7. LDE Acylation

Lipid-derived electrophiles (LDEs) refer to the reactive lipid metabolites generated by lipid peroxidation or other metabolic pathways [103]. Endogenous accumulation of oxidized lipid products has been reported as a biomarker of oxidative stress [104]. LDEs include acrolein, malonaldehyde (MDA), 4-hydroxy-2-nonenal (4-HNE), 15-deoxy-D12, 14-prostaglandin J2 (14-PGJ2), and 2-*trans*-hexadecenal (2-HD). These lipid metabolites can form covalent adducts with nucleophilic residues of proteins, such as cysteine, lysine, and histidine, via Michael addition (e.g., α, β-unsaturated carbonyls) or irreversible Schiff-base formation (e.g., aldehydes) [72,73] in organisms (Table 1). More than 2300 proteins and 500 cysteine sites in cell lines have been reported to be targeted by acrolein [105]. In a process called γ-oxononanoylation, 4-oxo-2-nonenal (4-ONE) attaches to the lysine residues of histones [106], a modification that can be reversed by SIRT2 in organisms [107].

## 3. Detection of Protein Lipidation

Detection of lipidated proteins involves challenging steps, including enrichment to identify the modification type and site, stoichiometric quantitation of the modification, and visualization of the modified protein. Despite these limitations, significant progress in the characterization of lipidated proteins has been made in the past few years.

The common “bottom up” high-throughput proteomics is considered a suitable approach to address these challenges through enrichment and digestion, multi-dimensional chromatographic separation, and high-throughput mass spectrometry detection [108,109,110,111]. Although MS-based detection approaches are highly sensitive in identifying lipidated proteins and modification sites, these approaches often require specialized protein enrichment methods, where is a filed hard to break through. Especially, for some very hydrophobic lipidated proteins, the common “bottom up” proteomics tends to underrepresent them. Thus, some studies have focused on detecting hydrophobic proteins with specific MS technique. Among them, the group of Robinson [112], who created a new technology—gas-phase structural biology MS to study hydrophobic proteins and protein-lipid interactions [113], while still deficient for high-throughput detection of lipidated proteins [114].

### 3.1. Qualitative Methods

#### 3.1.1. Radioactive Isotope-Labeling

**Figure 1:**
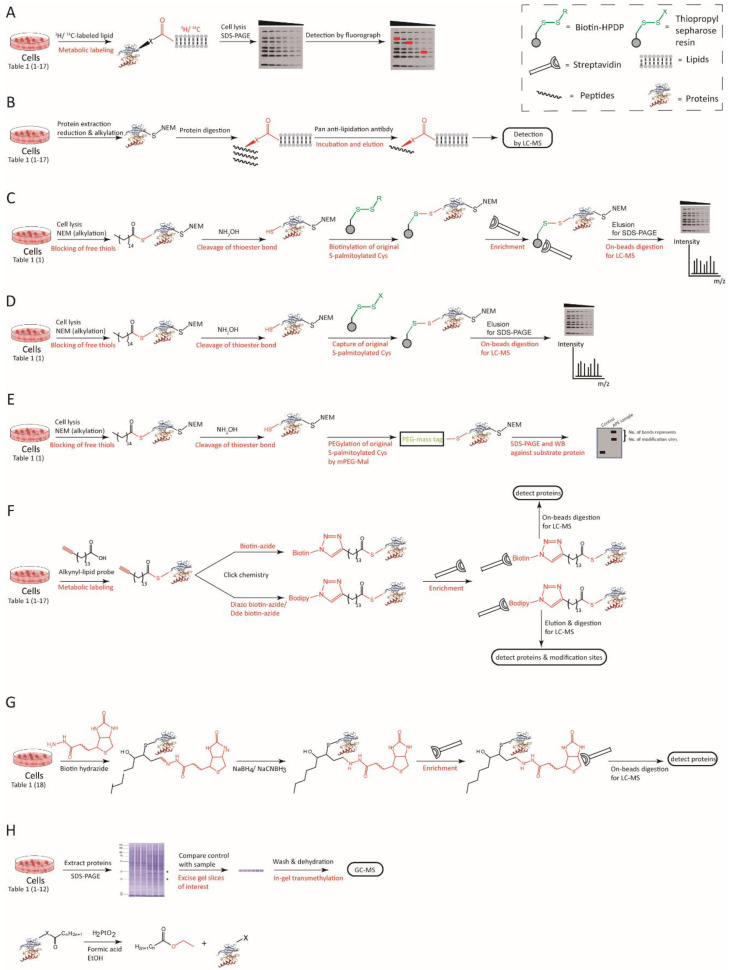
Various analytical methods to identify and characterize protein lipidations. (**A**) Radioactive isotope-labeling. Any type of protein lipidation can be identified using this method if the corresponding isotope-labeled lipid is available; (**B**) Antibody affinity enrichment. In general, any type of protein lipidation can be detected if a suitable pan-antibody is available; (**C**–**E**) ABE and similar methods. These methods are used for detecting S-pamitoylation; (**F**) Click chemistry. Protein lipidations that can react with specific alkynyl/azide-lipid probes can be identified; (**G**) Biotin hydrazide affinity capture. Only proteins containing carbonyl or aldehyde groups are suitable for this method to detect the LDE modifications; (**H**) Lipid esterification. Some saturated or unsaturated fatty acid moieties derived from protein acylations can be identified if the process of esterification on dissociative lipid (usually hydrolysis) is available.

Traditionally, metabolic incorporation of radiolabeled lipids is used to identify protein fatty acylation and prenylation [26,115,116,117]. For instance, incorporation of radioisotope-labeled palmitic acid is used as the gold standard for identifying S-palmitoylation [25,26,117,118]. In this strategy, ^3^H/^14^C-labeled palmitic acid is added into the cell culture. The palmitic acid is then metabolically converted to palmitoyl-CoA, which attaches to a cysteine residue on substrate proteins via a thioester bond. The S-palmitoylated proteins are then detected via western blot (WB) followed autoradiography (Figure 1A). This method does not alter the structure of fatty acid moieties. However, it is time-consuming, relatively low in sensitivity, unsuitable for high-throughput screening, and poses safety and environmental risks [119] (Table 2).

#### 3.1.2. Antibody Affinity Enrichment

A few studies have used fatty-acyl–specific antibodies to analyze lipidated proteins. In these studies, modified proteins were affinity-purified and then identified through WB or MS (Figure 1B). Palmitoylated transitional endoplasmic reticulum ATPase [120] was identified using a pan anti-palmitoyl antibody, but this antibody has not been used in any other study yet. Using an anti-lysine 2-hydroxyisobutyrylation (Khib) antibody, 2-hydroxyisobutyrylated histone [121] was identified, and a commercial antibody of the same nature was used in later studies [122,123,124]. Although antibody-based approaches enable easy and convenient enrichment of the targeted modified proteins, pan antibodies that recognize specific lipidated proteins are difficult to generate (Table 2).

#### 3.1.3. Acyl-Biotin Exchange (ABE)

ABE was proposed in 2004 by the Drisdel group [125] to exclusively detect S-acylation of cysteine residues. This method is based on the high sensitivity of thioester bonds to weak bases such as hydroxylamine (NH_2_OH). In this method, free thiols on the cysteine residues of proteins are first blocked with N-ethylmaleimide (NEM). Next, the thioester bonds of S-palmitoylated cysteine residues are broken using NH_2_OH, and then the newly exposed thiols are captured with the biotinylated probe biotin-N-[6-(biotinamido)hexyl]-3′-(2′-pyridyldithio) propionamide (Biotin-HPDP). Afterward, S-acylated proteins are purified using streptavidin-conjugated agarose beads and identified using WB or proteins digested into peptides are subjected to LC-MS (Figure 1C). Using this approach, hundreds of S-palmitoylated proteins have been identified [13,35,126].

In the case of the acyl-resin–assisted capture (acyl-RAC) method, the biotinylated probe is replaced with a thiopropyl sepharose resin [127] (Figure 1D). This effective strategy is more convenient than ABE.

Acyl-PEG exchange (APE) or acyl-PEGyl exchange gel shift (APEGS) is a mass-tag–labeling method to stoichiometrically evaluate endogenous levels of S-acylated proteins [128]. After liberating acylated cysteines by using NH_2_OH, free thiols are tagged with PEG-N-ethylmaleimide to increase the mass of each S-palmitoylated protein by adding a pre-defined PEG linker, whereby S-palmitoylated proteins can be distinguished from non-acylated proteins (Figure 1E). The shift in mass (e.g., 5 or 10 kD) is easily detectable via SDS-PAGE/WB without further enrichment. Furthermore, researchers can easily determine the number of S-acylated sites or quantify the ratio of unmodified proteins to S-acylated proteins. The APE method, however, is difficult to scale up for high-throughput analyses.

All the three acyl-exchange methods mentioned above require complete blockage of the reduced cysteine residues, efficient thioester hydrolysis, and thorough disulfide-exchange reactions to label and identify palmitoylated proteins. Furthermore, streptavidin-bead enrichment is associated with a high background signal. All these factors have resulted in significant numbers of false positives [129,130]. In addition, they cannot be generalized to detect other lipid modifications, such as isoprenylation (Table 2).

#### 3.1.4. Click Chemistry

Bio-orthogonal chemical probes include terminal alkyne or azido (ω-alkyne or ω-azido) lipid derivatives (fatty acids, sterols, and isoprenoids). The “click chemistry reaction” involves such probes and a highly efficient copper(I)-catalyzed cycloaddition reaction [131]. In this method, alkynyl-lipids are first metabolically incorporated. Next, the alkyne tag on the modified proteins is covalently attached to biotin-azide or a derivative through the click reaction. Subsequently, streptavidin beads are employed to pull down the proteins tagged with alkynyl-azide, and then these affinity-purified proteins digest into peptides are subjected to LC-MS to identify them and their modified sites (Figure 1F). In contrast to ABE, bio-orthogonal labeling in conjunction with the traditional pulse-chase method allows dynamic measurement of the rates of protein incorporation and turnover. Both alkyne- and azido-fatty acid probes have been developed for the click chemistry [132] and widely applied to the global analysis of N-myristoylated [133,134], S-palmitoylated [135,136], S-LDE–acylated [137,138], S-prenylated [15], cholesterolated [139], or monounsaturated-fatty-acid–modified [140] proteins.

Other click-based probes have also been developed. For example, the isoTOP-ABPP chemoproteomic platform has been used with an iodoacetamide-alkyne (IA-alk) probe and TEV-protease–cleavable biotin tags [103] to quantitate LDEs; an azido-biotin reagent has been used with a photocleavable linker (PC biotin-azide) [141,142] to analyze protein modification with electrophiles; and diazo biotin-azide [143] and 1-(4,4-dimethyl-2,6-dioxocyclohex-1-ylidene)ethyl (Dde) biotin-azide [144] have been used for high-throughput analyses. Alkynyl lipids with various chain lengths have been used to distinguish different types of protein lipidations, such as myristoylation, palmitoylation, stearoylation, prenylation [15], and monounsaturated fatty acylation [145] (Table 2).

#### 3.1.5. Biotin Hydrazide Affinity Capture

Carbonyl groups, as the feature groups of various proteins modified by LDEs, can react with hydrazides to form hydrazones and be promptly reduced by borohydride to generate stable secondary amines [146]. A biotin hydrazide affinity labeling and capture approach has been deployed to enrich and analyze HNE-adducted proteins [147] (Figure 1G). It is still unclear whether the carbonyl groups are generated by LDE-modification or protein oxidation in general. Moreover, this method also detects other carbonyl modifications as background signal (Table 2).

#### 3.1.6. Lipid Esterification

Lipid esterification methods mainly identify the lipid moieties in modified proteins through esterification of hydrolytically released lipid molecules, followed by gas chromatography–mass spectrometry (GC-MS) analysis. The integrated stable isotope-coded fatty acid transmethylation and mass spectrometry (iFAT-MS) method was developed to identify S- or O-acylated proteins [148]. In this method, proteins are extracted, quantified, and then resolved via SDS-PAGE. Subsequently, the gels are stained, and the protein bands are excised. The control and sample are transmethylated with d0- and d3-methanol, respectively. Derivatized fatty acids are analyzed using GC-MS (Figure 1H). iFAT-MS is an efficient approach to distinguish between N-, S-, and O-linked fatty acyl groups. S- and O-linkages, but not N-linkages, are cleaved via alkaline-catalyzed transmethylation. NH_2_OH treatment can then differentiate between the labile S-fatty acylation and resistant O-fatty acylation. Due to the relatively low efficiency of transesterification during the NH_2_OH treatment and poor separation of the esterified acyl moieties on GC, an alternative method, which replaces NH_2_OH with platinum (IV) oxide, has been devised [149] (Table 2).

Prenylated proteins can be examined via a similar approach, in which the double bonds on prenyl groups are first reduced through hydrogenation catalyed by platinum (IV) oxide, and then the prenyl moieties are released by Raney nickel cleavage. The reduced farnesyl and geranylgeranyl groups that are released are detected as 2,6,10-trimethyldodecane and 2,6,10,14-tetramethylhexadecane, respectively [150].

#### 3.1.7. Bioinformatics Tools

To consolidate the data from reports on S-palmitoylation and provide proteomic profiling resources, an online database named SwissPalm (http://swisspalm.epfl.ch/, accessed on 2 February 2022) has been created [32,33]. Release III of this database comprises 12688 palmitoylated proteins and 7459 palmitoylated sites, derived from 1198 studies in 68 species. It provides a user-friendly platform for researchers to retrieve proteins of interest, to assist and decide whether a specific protein may be S-palmitoylated, to predict potential S-palmitoylation sites, and to identify orthologues and potential functions. In addition, NBA-Palm (http://nbapalm.biocuckoo.org, accessed on 2 February 2022) [151], CSS-Palm 1.0/2.0 (http://csspalm.biocuckoo.org, accessed on 2 February 2022) [152,153] and CKSAAP-Palm (https://omictools.com/cksaap-palm-tool, accessed on 2 February 2022) [154] are other available programs to predict palmitoylated proteins and sites (Table 2).

### 3.2. Quantitative Proteomics Methods

Using the above discussed MS approaches and tag-enrichment strategies, various lipidated proteins from a wide range of organisms have been identified. However, these datasets often contain a large number of false positives. By using quantitative chemical proteomics, lipidated proteins can be quantified with high-confidence, based on signal-to-noise ratio (SNR), spectral counting, signal intensity, and qualify P-value or false discovery rates (FDR) value [36,79,134,155,156].

#### 3.2.1. Stable Isotope Labeling with Amino Acids in Cell Culture (SILAC)

In SILAC, cells are grown in media lacking certain essential amino acids but supplemented with isotopically labeled or unlabeled ones. Proteins from the test and control samples are then equally mixed and subjected to MS to quantify and identify the peptides with labeled or unlabeled amino acids [157]. Using SILAC and 17-octadecynoic acid (17-ODYA) bio-orthogonal labeling, 415 high-confidence palmitoylated proteins have been identified, and by including a pulse-chase method, a global quantitative map of dynamic protein palmitoylation events has been generated [36]. Using a combination of ABE and Stable Isotope Labeling of Mammals (SILAM), the S-palmitoylated protein profile of the glial cells from a mouse model of Huntington’s disease has been characterized [158], and 151 high-confidence differentially palmitoylated proteins have been identified using the cysteine-stable isotope labeling (Cysteine-SILAC) method [159]. In this method, mass tags are incorporated to cysteine residues, and discriminated in MS with pairs (if two tags are used) or even triplets (if three). Hence the co-elution feature of the peptide isotope pairs improves confidence in their identification.

#### 3.2.2. In Vitro Isotope Labeling

To profile the intrinsic reactivity of cysteine residues quantitatively, an approach named “isotopic tandem orthogonal proteolysis-activity-based protein profiling” (isoTOP-ABPP) has been described [160]. In this approach, an electrophilic alkynylated iodoacetamide (IA) probe is used to tag the cysteine residues in native proteins. The alkynyl group in IA conjugates the probe via the click chemistry to an azide-functionalized, isotopically labeled TEV-protease recognition peptide containing a biotin group. Finally, the tagged proteins are purified using streptavidin-conjugated beads and then quantified via MS. Although the isoTOP-ABPP method has been designed to quantitate reactive cysteines, it can be adapted to quantitate lipidated proteins. For instance, LDE modification of cysteines was evaluated using this approach [103]. Isobaric tagging for relative and absolute quantification/isobaric tandem mass tags (iTRAQ/TMT) [125,161] and iodoacetyl isobaric tandem mass tags (iodoTMT) [162] have also been reported.

### 3.3. Dynamic Visualization Methods

In addition to direct quantification, an azido fluorescence tag can be attached to specific lipidated proteins to achieve in-gel visualization by using the click chemistry approach [133]. To date, various subcellular localizations of lipidated proteins have been observed using ω-alk fatty acids [132]. Live-cell imaging of S-palmitoylated proteins has been achieved by using this bio-orthogonal strategy [163]. Additionally, imaging of global prenylated proteins by using fluorescent analogues of farnesyl and geranylgeranyl pyrophosphates has been reported [164]. In situ proximity ligation assay (PLA) alongside fluorescent imaging based on alkynyl fatty acids has been applied to track lipidated proteins with high spatial resolution in live cells [163,165].

## 4. Detection of PUFA-Modified Proteins

In Section 2, we reviewed various protein lipidations and discussed hydrophobic modification as a universal process that can regulate many fundamental biological functions. Decades ago, it was suggested that proteins can be acylated with arachidonic acid (C20:4n6) and eicosapentaenoic acid (20:5n3) [61]. Surprisingly, few studies have since reported on PUFA acylation. Only one study has described the arachidonoyl modification on the N-terminus of lens fiber major intrinsic protein (AQP0) [166]. Possible reasons for the scarcity in publication, limitations in current methodology, and potential approaches to detect PUFA-modified proteins are discussed below.

### 4.1. Difficulties in Detecting PUFA-Modified Proteins

Humans are estimated to express 20,000 proteins [167,168] (https://www.hupo.org/human-proteome-project/, accessed on 2 February 2022), and PTMs of proteins play vital roles in diverse biological processes. Protein lipidation accounts for a small fraction of PTMs and of which PUFA modification represents the minority. Therefore, detection of the low levels of PUFA-modified proteins is challenging. Furthermore, double bonds in fatty acids are relatively unstable and PUFAs, especially ω-3 PUFA, are prone to peroxidation [169,170]. The peroxidation of PUFAs may happen in vivo as a part of the biological process or in vitro during sample enrichment processes in practice.

Saturated acyl chains can tightly pack with cholesterol to form ordered microdomains, such as membrane rafts, whereas unsaturated acyl chains do not pack well with cholesterol and thus form a disordered, liquid phase [171,172]. Unlike saturated fatty acids, PUFAs can target proteins to various microdomains and require diverse protein extraction procedures due to their structural complexity.

### 4.2. Limitations in Current Methodology

Concurrent accurate detection of abundant and scarce proteins via MS-based, high-throughput proteomic analyses is challenging [173,174,175]. Although it is feasible to use the ABE method for the enrichment of PUFA-modified proteins, this method is specific for thioester-bond analysis, and PUFA modification of proteins through other bonds cannot be detected. Detection of LDE-modified proteins was discussed in Section 2.7. It is important to distinguish proteins directly modified with LDEs from those initially modified by PUFAs and then oxidized. However, there is currently no method available for this purpose.

### 4.3. Potential Solutions

**Figure 2:**
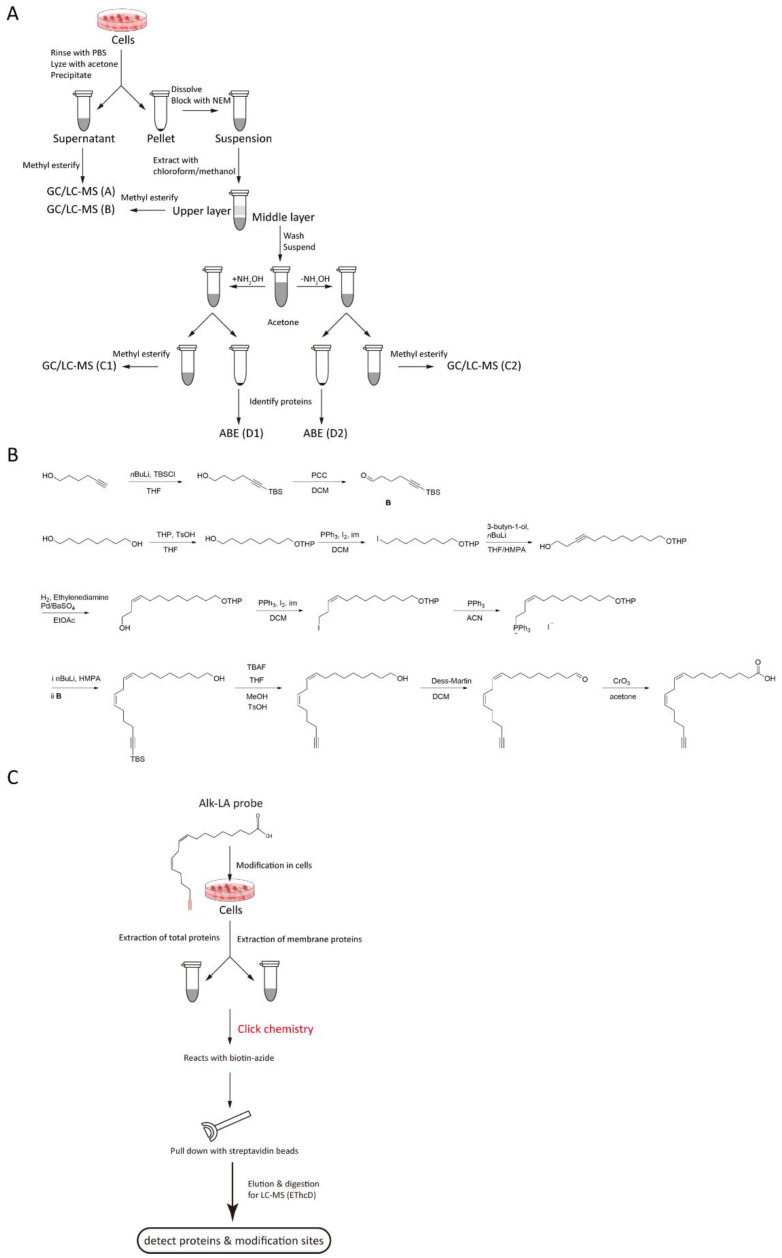
Methods to detect PUFA-modified proteins. (**A**) Flowchart of ABE and GC/LC-MS. Group A treats the supernatant from the acetone precipitation in cells; Group B treats the precipitation from the acetone precipitation in cells; Group C1 and D1 (+NH_2_OH group) add NH_2_OH and acetone to the above precipitation and further treats the second supernatant and precipitation as group C1 and D1; Group C2 and D2 (-NH_2_OH group) add control and acetone to the above precipitation and further treats the second supernatant and precipitation as group C2 and D2; (**B**) The synthesis of the alkynyl-linoleic acid (alk-LA) probe. (**C**) Flowchart of the Click-chemistry method employed on total-protein or membrane-protein samples.

To characterize PUFA-modified proteins, a method that combines ABE and methyl esterification PUFA to GC/LC-MS detection and thereby detects lipidated proteins and simultaneously analyzes their fatty acid moieties is better [150,176,177]. In this method, cells are washed with PBS and then lyzed with acetone. Afterward, proteins are precipitated, and the free fatty acid content of the supernatant is characterized (GC/LC-MS A). The protein pellet is suspended, and free thiol groups are blocked with NEM. The sample is then subjected to lipid extraction with chloroform/methanol, and the free fatty acid content of the upper layer is characterized (GC/LC-MS B). The proteins in the middle layer are resuspended, divided into two, and then treated with or without NH_2_OH. The supernatant and pellet are used for the characterization of the fatty acids (GC/LC-MS C) and identification of the modified proteins (LC-MS D), respectively (Figure 2A).

The above ABE/GC-MS method is specific to S-fatty acylated proteins. To detect other potential PUFA-protein linkages, we propose a synthesized alkynyl-linoleic acid (alk-LA) probe (Figure 2B) in light of the synthetic method of alkynyl-palmitic acid (alk-PA) probe [178] and used the click-chemistry method for high-throughput detection of LA-modified proteins. In this strategy, the cells are incubated with the alk-LA probe for 24 h. Then, total proteins and membrane proteins are extracted, followed by the click-chemistry reaction. Afterward, the modified proteins are pulled down using streptavidin beads, digested, and finally analyzed via LC-MS (Figure 2C) [140].

In recent years, a novel electron-transfer/higher-energy collision dissociation (EThcD) approach that preserves the original reporter ion channels and mitigates bias against the low-charge states has been proposed and optimized systematically [179,180]. This method significantly improves data quality in quantitative proteomics and proteome-wide PTM studies [181]. We think that this approach can yield a higher throughput in detecting the above-mentioned LA-modified proteins than the HCD approach (Figure 2C). However, the general problem with “bottom up” proteomics is that some tryptic peptides are just not suitable for identification or “bad flyers”, i.e., low ionization efficiency/suppression, although many solutions have been proposed, such as the EThcD, ion-mobility spectrometry (IMS) (which as a further dimension for MS analysis) [182,183], and using complementary digestion enzymes to improve sequence coverage. IMS separates ions with different conformations and charge states by guiding them through buffer gas under electric fields [184]. In recent years, researchers utilize IMS combining MS to carry out high-throughput proteomics, by this way, samples can be analyzed based on both structure and *m*/*z* to improve detection throughput. Because the IMS is much faster than LC, IMS can be inserted between LC and MS for an additional separation dimension to improve protein coverage without sacrificing the overall duty cycle/throughput [185].

Another approach is the using of “top down” proteomics to identification lipidation by MS of intact proteins. A number of intact proteins recognition technique have emerged in recent years [186], and further fueled by increase in biosimilars [187]. Generally, increased peak capacity with advanced packing material as well as longer separation columns or integrating IMS significantly improves performance in “top down” and “bottom up” proteomics [186,188].

It is noteworthy that the alk-LA probe click chemistry method also effectively distinguishes PUFA-modification from LDE-modification since only LA-acylated proteins can be pulled down in this method. To minimize the peroxidation of the LA moiety of modified proteins, the samples should be supplemented with antioxidants.

In addition, whether PUFA-acylated proteins are tethered onto cellular membranes can be determined. For this purpose, the membrane fraction of the samples should be enriched first. PUFA-modified proteins may be concentrated by taking advantage of their double-bonded feature.

## 5. Conclusions

In this review, we provided potential approaches to detect PUFA-modified proteins. Nevertheless, much remains to be explored. For instance, it is unclear how many proteins can be PUFA-lipidated and under what circumstances; what functionality PUFA modification confers to proteins; and whether ω3 and ω6 PUFA modifications differ in functionality. We hope that this review will generate interest in the research community to further study protein lipidation.

## Figures and Tables

**Table 1:** Types of cellular protein lipidation.

Modification		Lipid	Structure	Linkage	Modified Residue	References
1	S-palmitoylation	Palmitic acid (C16:0)	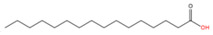	Thioester	Cysteine	[22,23,28,29]
2	N-terminal palmitoylation	Palmitic acid (C16:0)		Amide	N-terminal Cysteine	[31,44]
3	N^ε^-palmitoylation	Palmitic acid (C16:0)		Amide	Lysine	[45,46]
4	O-palmitoylation	Palmitic acid (C16:0)		Oxyester	Serine	[47]
					Threonine	[48]
5	N-terminal myristoylation	Myristic acid (C14:0)	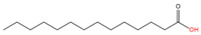	Amide	N-terminal Glycine	[49]
6	N^ε^-myristoylation	Myristic acid (C14:0)		Amide	Lysine	[50,51,52]
7	S-stearoylation	Stearic acid (C18:0)	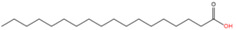	Thioester	Cysteine	[53,54]
8	O-octanoylation	Octanoic acid (C8:0)	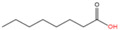	Oxyester	Serine	[55,56]
9	O-palmitoleoylation	Palmitoleic acid (C16:1n7)	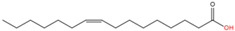	Oxyester	Serine	[57,58,59]
10	N-oleoylation	Oleic acid (C18:1n9)	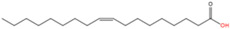	Amide	Lysine	[60]
11	Unnamed	Arachidonic acid (C20:4n6)	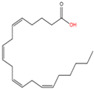	Yet unknown	Yet unknown	[61]
12	Unnamed	Eicosapentaenoic acid (C20:5n3)	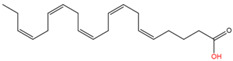	Yet unknown	Yet unknown	[61]
13	N-lipoylation	Lipoic acid	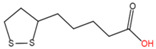	Amide	Lysine	[62,63]
14	S-prenylation	Isoprenoid	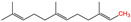	Untitled	C-terminal Cysteine	[64,65]
			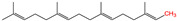			
15	C-terminal phosphatidyl-ethanolaminylation	PE	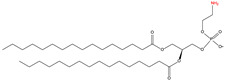	Amide	C-terminal Glycine	[66,67]
16	C-terminal cholesterolyation	Cholesterol	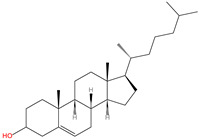	Oxyester	C-terminus	[68,69]
17	C-terminal GPI anchor	GPI	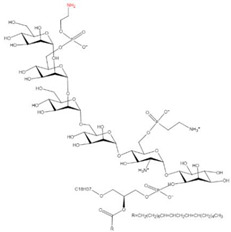	Amide	C-terminus	[70,71]
18	LDE acylation	LDE	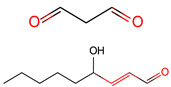	Carbonyls	Nucleophilic residues	[72,73]
				Aldehydes		

N-system nomenclature was used for the fatty acids (the order of carbon atoms starts from the methyl carbon of the fatty acid).

**Table 2:** Well-established enrichment methods to assess for protein lipidation.

	Radioactive Isotope-Labeling	Antibody Affinity Enrichment	ABE	Click Chemistry	Biotin Hydrazide Affinity Capture	Lipid Esterification
Procedures	^3^H/^14^C metabolic labeling, radiography	Pan-antibody detection of modified moieties	Block-free thiols, cleavage thioester bonds, capture-exposed thiols, IP with streptavidin, WB or elution for MS	alkynyl/azide-lipid probe incorporation, click reaction, IP with streptavidin, elution for MS	Carbonyl group and biotin-hydrazide linkage, capture and analyze LDEs	Dissociative lipids with esterification, GC-MS analysis
Applications	Detection of lipidated proteins	Detection of lipidated proteins	Detection of Cysteine S-acylation	Detection of lipidated proteins	Detection of protein lipidation with LDEs	Detection of lipidation
Advantages	Direct detection of lipidated proteins without altering the lipid structure	Amenable for protein enrichment	Efficiently distinguishes S-palmitoylation	Availability of alkynyl/azide-lipid probes	Simple method for LDE detection	Quantification of lipid species
Disadvantages	Radioactive exposure, limited by the availability of radio-labeled fatty acid	Limited by the availability of pan-antibodies	High background	Interference with endogenous lipidation	Unable to identify the modified sites, high background	Unable to identify the modified sites, high background
Throughput	Low	High	High	High	High	High

## Data Availability

Not applicable.

## Abbreviations

14-PGJ2	15-Deoxy-D12, 14-prostaglandin J2
17-ODYA	17-Octadecynoic acid
2-HD	2-*trans*-Hexadecenal
4-HNE	4-Hydroxy-2-nonenal
4-ONE	4-Oxo-2-nonenal
ABE	Acyl-biotin exchange
acyl-RAC	Acyl-resin–assisted capture
AKAP12	A-kinase anchoring protein 12
alk-LA	Alkynyl-linoleic acid
alk-LDEs	Alkynyl analogs of LDE
alk-PA	Alkynyl-palmitic acid
APE	Acyl-PEG exchange
APEGS	Acyl-pegyl exchange gel shift
APT	Acyl protein thioesterase
AQP0/MIP	Major intrinsic protein of lens fiber
BAX	BCL2-associated X, apoptosis regulator
BCKDHA	Branched-chain keto acid dehydrogenase E1 subunit alpha
Biotin-HPDP	Biotin-N-[6-(biotinamido)hexyl]-3′-(2′-pyridyldithio) propionamide
CD	Circular dichroism
Cysteine-SILAC	Cysteine-stable isotope labeling
Dde	1-(4,4-Dimethyl-2,6-dioxocyclohex-1-ylidene)ethyl
EP300	E1A-binding protein p300
EThcD	Electron-transfer/higher-energy collision dissociation
FAS	Fas cell surface death receptor
FASN	Fatty acid synthase
FKBP4	Peptidyl-prolyl cis-trans isomerase FKBP4
FTase	Farnesyltransferase
FXR2	FMR1 autosomal homolog 2
GC-MS	Gas chromatography–mass spectrometry
GCV	Glycine cleavage system
GGTase-I	Geranylgeranyltransferase type I
GGTase-II	Geranylgeranyltransferase type II
GOAT	Ghrelin O-acyltransferase
GPI	Glycosylphosphatidylinositol
HH	Hedgehog family
HK1S	Hexokinase 1 variant in mammalian spermatozoa
HRAS	HRas proto-oncogene, GTPase
IA-alk	Iodoacetamide-alkyne
iFAT-MS	Isotope-coded fatty acid transmethylation–mass spectrometry
IMS	Ion-mobility spectrometry
iodoTMT	Iodoacetyl isobaric tandem mass tag
IP	Immunoprecipitation
isoTOP-ABPP	Isotopic tandem orthogonal proteolysis–activity-based protein profiling
ITPR1	Inositol 1,4,5-triphosphate receptor type I
iTRAQ/TMT	Isobaric tag for relative and absolute quantitation/isobaric tandem mass tag
JAM3	Junctional adhesion molecule 3
KDH	α-Ketoglutarate
Khib	Lysine 2-hydroxyisobutyrylation
KRAS	KRas proto-oncogene, GTPase
LC3	Microtubule-Associated Protein 1 Light Chain 3 Alpha
LC-MS	Liquid Chromatography-Mass Spectrometry
LDE	Lipid-Derived Electrophile
LPCAT1	Lysophosphatidylcholine Acyltransferase 1
MDA	Malonaldehyde
MS	Mass Spectrometry
NAT	N-terminal acetyltransferase
NEM	N-ethylmaleimide
NH_2_OH	Hydroxylamine
NMR	Nuclear Magnetic Resonance
NMT	N-myristoyl transferase
NRAS	NRas proto-oncogene, GTPase
PAT	Palmitoyl acyltransferase
PC	Photocleavable
PDH	Pyruvate dehydrogenase
PE	Phosphatidylethanolamine
PI-PLC	Phosphatidylinositol-specific phospholipase C
PLA	Proximity ligation assay
PRDX6	Peroxiredoxin-6
PRKAB1	Protein kinase AMP-activated non-catalytic subunit beta 1
PTM	Post-translational modification
PUFA	Polyunsaturated fatty acid
SFA	Saturated fatty acid
SILAC	Stable isotope labeling with amino acids in cell culture
SILAM	Stable isotope labeling of mammals
SIRT	Sirtuin
SNR	Signal-to-noise ratio
SRC	SRC proto-oncogene, non-receptor tyrosine kinase
VIM	Vimentin
WB	Western blotting
